# Buckling Behavior Analysis of Kirigami Structure Under Tension

**DOI:** 10.3390/mi15111398

**Published:** 2024-11-20

**Authors:** Pengzhong Dai, Ziqi Li, Xiaoyang Zhang, Qingmin Yu

**Affiliations:** 1 School of Aeronautics, Northwestern Polytechnical University, Xi’an 710072, China; daipengzhong@mail.nwpu.edu.cn; 2 National Key Laboratory of Aircraft Configuration Design, Xi’an 710072, China; 3 School of Mechanics, Civil Engineering and Architecture, Northwestern Polytechnical University, Xi’an 710129, China; ziqili@mail.nwpu.edu.cn (Z.L.); zhangxiaoyang123@mail.nwpu.edu.cn (X.Z.)

**Keywords:** kirigami structure, buckling behavior, stretchability

## Abstract

Flexible electronic technology has attracted great interest, where rigid and brittle semiconductor materials can withstand large deformation. In order to improve the stretchability of devices, many novel structures have been designed, such as the classical “wavy” structure, the island-bridge structure, and origami structures that achieve stretchability through creases. However, the stretchability of these structures is still not large enough. Inspired by traditional kirigami, the stretchability of devices is achieved by making various periodic cuts in the substrate while the devices are placed in the area around the cuts. The previous research mainly focused on the change in the electrical properties of the structure during the deformation process, and there were few studies on the mechanical mechanisms. Therefore, this paper studies the buckling behavior of the kirigami structure when the substrate is stretched, and its mechanism can provide guidance for practical applications.

## 1. Introduction

In recent years, the field of flexible electronics has achieved significant progress, making it a very promising technology. It has broad applications and can be applied to many emerging devices, such as solar cells [1], implantable devices [2], medical monitoring equipment [3,4,5], eye cameras [6,7], flexible displays [8,9], and flexible sensors [10,11]. Electronic devices may be subjected to external deformation such as stretching, bending, and twisting. However, there are still challenges to achieve the optimal stretchability of devices while maintaining their initial performance under large external loadings. In order to solve the above contradiction, many novel mechanical structures were proposed such as island-bridge structures [12] and micro-patterned structures [13]. Additionally, structures inspired by the ancient art of kirigami have attracted extensive research and attention recently. For example, they are used in tensegrity structures [14], flexible EHD pumps [15], and flexible energy harvesters [16]. Because the kirigami structure is easy to stretch, has a high cyclic life, and a high stretchability, it has been applied to various emerging flexible electronic systems. When the kirigami structure is stretched, the applied tensile stress is converted into torsional stress through specific points between the cuts to achieve the stretchability of the device. The device is little affected during the deformation process, and its electrical properties remain nearly unchanged. Additionally, kirigami structures can also be cut, bent, and folded to make different three-dimensional structures, and can even achieve extreme deformation and transition from 2D to 3D structures by out-of-plane buckling [17,18,19].

There are few studies on the mechanical behaviors of kirigami structure in previous articles. In this paper, the buckling behavior of the structure is systematically studied through the combination of theory and finite element simulation. By establishing the proportional law of local strain, the potential relationship between the buckling behavior of structure (including maximum strain, stretchability, maximum out-of-plane displacement, and critical buckling load) and geometric parameters (including cut length, cut width, cut spacing, number of cuts, and thickness of substrate) is studied when the structure is stretched. The theory and simulation show that the kirigami structure can effectively improve the deformation ability and significantly reduce the stress of electronic devices through out-of-plane deformation. The results in this paper can provide effective guidance for the design of electronic devices.

## 2. Materials and Methods

### 2.1. Kirigami Structure

**Figure 1:**
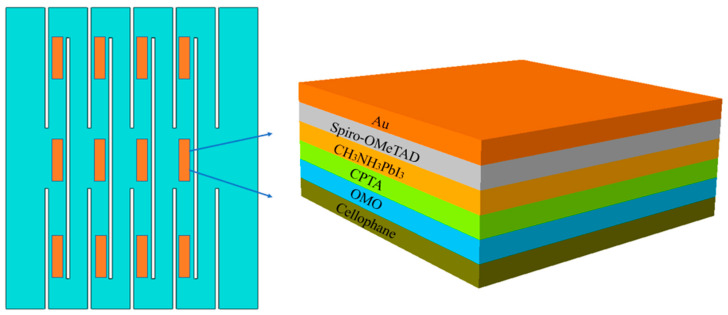
The schematic diagram of highly stretchable perovskite solar cell based on kirigami structure.

The schematic diagram of highly stretchable perovskite solar cell based on kirigami structure is shown in Figure 1. When the applied strain is small, the stress in the structure is small and evenly distributed; when the applied strain further increases, the stress near the cut end will be higher than that in other areas. If the functional layer of electronic devices is located near the cut end, high stress may affect the performance of solar cells. Therefore, devices will be placed far away from the end of the cut to reduce the impact of high stress on the performance of devices.

**Figure 2:**
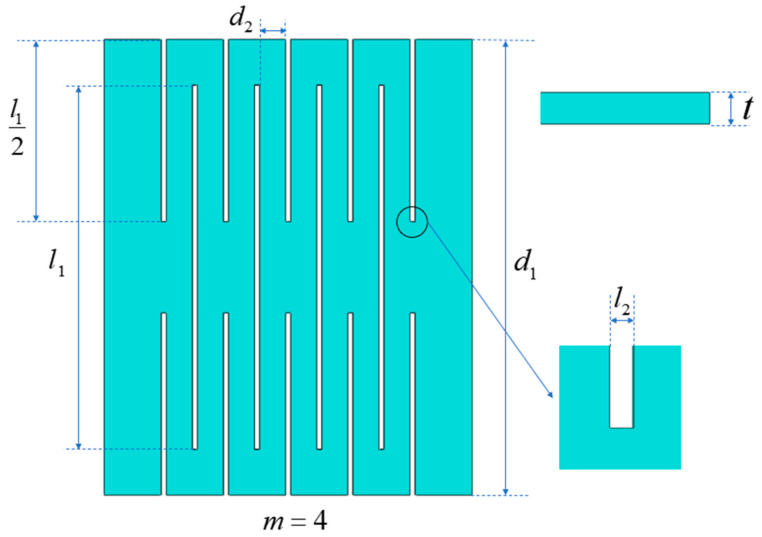
Schematic diagram of key parameters of kirigami structure.

This paper takes ultra-thin perovskite solar cells (PSCs) based on cellophane substrate as an example to study the effect of geometric parameters on the buckling behavior of kirigami structure. In order to simplify the analysis, cellophane substrate was selected as the research platform, where periodic cuts are fabricated. The diagram of the kirigami structure is shown in Figure 2. The number of periodic cuts in the figure is m=4, the length of the cut is $l_{1}$, the spacing between the cuts is $d_{2}$, the width of each cut is $l_{2}$, the width of the substrate is $d_{1}$, and the thickness of the substrate is *t*. The results show that the geometric parameters of the kirigami structure can be characterized by five dimensionless parameters, i.e., the number of units *m*, the normalized cuts length $l_{1}/d_{1}$, the normalized cuts spacing $d_{2}/d_{1}$, the normalized cut width $l_{2}/d_{1}$, and the normalized substrate thickness $t/d_{1}$.

### 2.2. Theory Analysis

During stretching, there will be periodic strips on the substrate due to the existence of cuts. Because the thickness of the strip is far less than the width of the strip, the pure in-plane bending deformation will produce large strain energy. Therefore, except for the left and right vertical strips connected at both ends, all unit strips have experienced the combined deformation of in-plane bending, out-of-plane bending, and torsion in the stretching process. Driven by the external force, its in-plane stiffness decreases, and the substrate based on the kirigami structure is transformed into a three-dimensional structure through bending and rotating strips. With the increase in applied strain, the strips tend to be parallel to the loading direction. From the stress–strain curve, it can be seen that the cellophane substrate with cuts has much higher stretchability than that without cuts [1]. For the non-cut substrate, the stress increases rapidly to tens of MPA when stretched; in contrast, the stress of the substrate with cuts is several orders of magnitude lower.

The stress–strain curve of the substrate with cuts can be divided into three stages [1,11,20,21]. In the first stage, when the applied strain is relatively small, the in-plane deformation can ensure the initial rigid state. The effective stiffness of the structure is controlled by the plane bending of the strip. The mechanical deformation behavior is similar to that of the not-cut substrate, mainly experiencing elastic deformation and in-plane tension. In the second stage, the strain of the structure will exceed the critical buckling value, and the in-plane deformation becomes unstable and begins to transform into out-of-plane buckling. The deformation of the structure changes from tension to bending and rotation of the strip. This out-of-plane deformation enables the strip to withstand large strain with very low stress. Compared with the not-cut substrate, the stress is reduced by more than 2 orders of magnitude, so as to obtain higher stretchability. In the third stage, with the further increase in the applied strain, the subsequent deformation changes from bending to tension, and the hardening of the material at the edge of the cuts will produce greater stress, resulting in a sharp increase in effective stiffness, and finally lead to the fracture of the devices. Since all strips begin to be parallel to the loading direction, the stress before the fracture of the substrate is almost unchanged.

The maximum strain in the kirigami structure generally appears in the connection area between adjacent vertical strips. It is a very important value to determine whether the structure fails and is the key to solving the stretchability of the kirigami structure. Therefore, it is necessary to study the maximum strain of kirigami structure when it is stretched. The in-plane and out-of-plane bending strain are linear with the cut spacing and the substrate thickness, respectively. In addition, the bending strain is independent of the cut width because it is usually much smaller than the strip width. Therefore, it can be assumed that the relationship between the maximum strain and the geometric parameters of the kirigami structure can be expressed as [20]

(1) $$\varepsilon_{\max}=\frac{d_{2}t}{{d_{1}}^{2}}F\left(\frac{l_{1}}{d_{1}},m,\varepsilon_{applied}\right)$$

where *F* is a dimensionless function related to the normalized cut length $l_{1}/d_{1}$, the number of periodic cuts *m* and the applied strain $\varepsilon_{applied}$, and the above relationship can be verified by finite element method.

In addition, according to the previous research on the buckling behavior of the serpentine conductor, the in-plane and out-of-plane bending strain are linearly proportional to the square and square root of the applied strain in the post-buckling process [18], i.e., ${\varepsilon_{applied}}^{2}$ and $\sqrt{\varepsilon_{applied}}$, which can also be applied to kirigami structure in this paper, verified by finite element method in the following. Therefore, the Equation (1) can be further expressed as [18]

(2) $$\varepsilon_{\max}=\frac{d_{2}t}{{d_{1}}^{2}}\left[g_{1}\left(\frac{l_{1}}{d_{1}},m\right){\varepsilon_{applied}}^{2}+g_{2}\left(\frac{l_{1}}{d_{1}},m\right)\sqrt{\varepsilon_{applied}}\right]$$

where $g_{1}$ and $g_{2}$ are dimensionless functions related to the normalized cut length $l_{1}/d_{1}$ and the number of cuts *m*, and $g_{1}$, $g_{2}$ can be solved by binary linear regression for geometric parameters, applied strain, and maximum strain.

### 2.3. Simulation Analysis

In order to verify the above theoretical model, this section uses the commercial finite element analysis software ABAQUS 6.14 to simulate the buckling process of kirigami structure under tension. In the finite element model, the kirigami structure is modeled as a reduced three-dimensional solid element (C3D8R). The elastic modulus of the substrate (cellophane) is 2200  MPa and the Poisson’s ratio is ν=0.4, assuming that the material is linearly elastic and isotropic.

In real life, this kirigami structure can increase the maximum strain that electronic devices can withstand. These devices using paper cutout structures are usually fixed at each end to an object that undergoes relative motion, i.e., the two ends of the device move relative to each other, so the left end of the substrate is fixed in the simulation and a displacement is applied to the right end. The magnitude of the applied displacement can be determined by $\varepsilon_{applied}=\Delta L/L$ where ΔL represents the applied displacement and *L* represents the length of the substrate. The finite element simulation process of kirigami structure includes two analysis steps. In the first step, the lowest buckling mode and critical buckling load are obtained by linear buckling analysis, and the displacement load is implemented as initial geometric imperfections in the second step to study the deformation of the kirigami structure under tension.

## 3. Results

### 3.1. Maximum Strain Versus Geometric Parameters

This section mainly studies the influence of geometric parameters of the kirigami structure on the maximum strain in the structure, including the width of each cut $l_{2}$, the spacing between the cuts $d_{2}$, the thickness of the substrate *t*, the length of the cut $l_{1}$, and the number of periodic cuts m.

**Figure 3:**
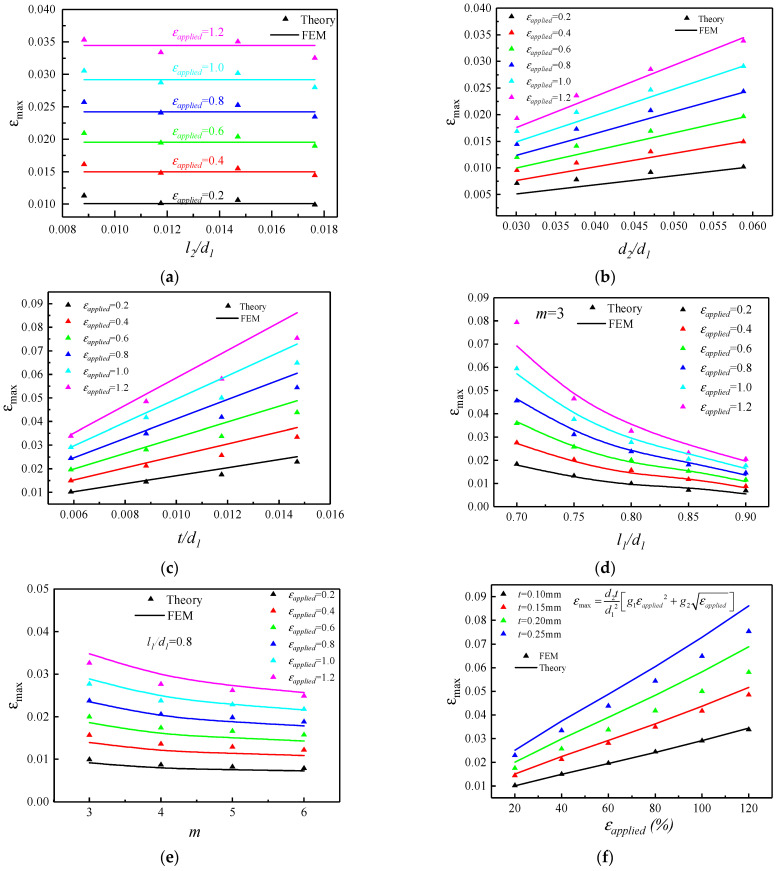
Theoretical and finite element results of maximum strain. (**a**) Normalized cut width, (**b**) normalized spacing between the cuts, (**c**) normalized thickness of substrate, (**d**) normalized cut length, (**e**) number of periodic cuts, and (**f**) applied strain. The baseline geometric parameters are $l_{2}=0.2\;\mathrm{mm}$, $d_{2}=1\;\mathrm{mm}$, t=0.1 mm, $l_{1}=13.6\;\mathrm{mm}$, and m=3, and the parameter in each figure varies in its representative range.

The effect of cut width on the maximum strain of the structure is shown in Figure 3a, selecting the dimensionless $l_{2}/d_{1}$ as the abscissa, where $l_{1}=13.6\;\mathrm{mm}$, $d_{1}=17\;\mathrm{mm}$, $d_{2}=1\;\mathrm{mm}$, m=3, and t=0.1 mm remains constant. It can be seen from the figure that for a certain applied strain, the maximum strain in the kirigami structure remains approximately unchanged as the width of the cut increases. The theoretical results are in good agreement with the finite element results. It can be concluded that during the stretching process of the kirigami structure, the change of the cut width has little effect on the maximum strain and can be ignored.

The maximum strain in the structure versus spacing between the cuts and thickness of the substrate are shown as Figure 3b,c, respectively. It can be seen from the figure that for a certain applied strain, with the increase in the spacing between the cuts and thickness of the substrate, the maximum strain in the kirigami structure will also increase. The relationship is approximately linear, the intercept of the straight line can approximately be regarded as 0, and the Equation (1) can be further verified. Additionally, the effect of the length of the cut and the number of periodic cuts on the maximum strain can be seen from Figure 3d,e, respectively. It can be concluded that as the length of the cut and the number of periodic cuts increases, the maximum strain in the kirigami structure decreases, where the relationship between maximum strain and geometric parameters is no longer approximately linear and the maximum strain decreases more and more slowly as the length of the cuts and the number of periodic cuts increase. Finally, the maximum strain in the structure was studied as applied strain increases for fixed geometric parameters as shown in Figure 3f, which can be used to prove the Equation (2) is correct.

### 3.2. Elastic Stretchability Versus Geometric Parameters

This section mainly studies the influence of the geometric parameters of the kirigami structure on the elastic stretchability. The elastic stretchability of the structure means that after releasing the applied strain, the structure can return to the state before stretching. Assuming that the yield strain of the substrate material is $\varepsilon_{yield}$, when the maximum strain $\varepsilon_{\max}$ in the kirigami structure reaches its yield strain, the structure is considered to have reached the limit of elastic stretching. Then, the expression can be obtained as [18]

(3) $$\frac{d_{2}t}{{d_{1}}^{2}}\left[g_{1}\left(\frac{l_{1}}{d_{1}},m\right){\varepsilon_{applied}}^{2}+g_{2}\left(\frac{l_{1}}{d_{1}},m\right)\sqrt{\varepsilon_{applied}}\right]=\varepsilon_{\mathrm{yield}}$$

Suppose that $\varepsilon_{applied}=\varepsilon_{stretchability}$, for the convenience of solving, the square root of elastic stretchability is denoted as $\lambda =\sqrt{\varepsilon_{stretchability}}$, and substituting it into Equation (3) can get the quartic equation about λ, which can be expressed as

(4) $$g_{1}\left(\frac{l_{1}}{d_{1}},m\right)\lambda^{4}+g_{2}\left(\frac{l_{1}}{d_{1}},m\right)\lambda -\frac{{d_{1}}^{2}\varepsilon_{yield}}{d_{2}t}=0\;(\lambda >0)$$

where $g_{1}$, $g_{2}$ can be solved by binary linear regression as mentioned above. The yield strain of the cellophane substrate is 5.5% and further solving the equation can obtain the value of λ (only the positive solution is taken), from which the solution of the elastic stretchability of the kirigami structure can be obtained as $\varepsilon_{stretchability}=\lambda^{2}$.

**Figure 4:**
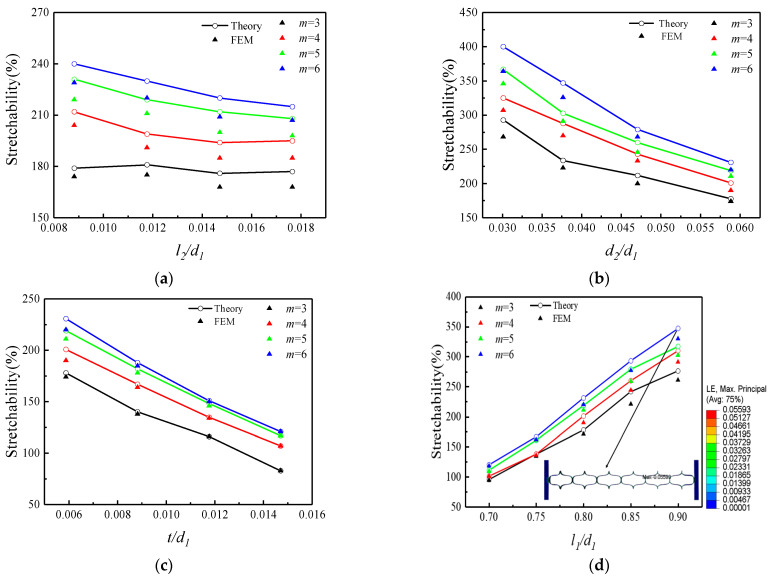
Theoretical and finite element results of elastic stretchability. (**a**) Normalized cut width, (**b**) normalized spacing between the cuts, (**c**) normalized thickness of substrate, and (**d**) normalized cut length. The baseline geometric parameters are $l_{2}=0.2\;\mathrm{mm}$, $d_{2}=1\;\mathrm{mm}$, t=0.1 mm, $l_{1}=13.6\;\mathrm{mm}$, and the parameter in each figure varies in its representative range.

The effect of cut width on the elastic stretchability of the structure is shown in Figure 4a, selecting the dimensionless $l_{2}/d_{1}$ as the abscissa, where $l_{1}=13.6\;\mathrm{mm}$, $d_{1}=17\;\mathrm{mm}$, $d_{2}=1\;\mathrm{mm}$, and t=0.1 mm remain constant. It can be seen from Figure 4a, the elastic stretchability of the kirigami structure decreases with increasing cut width, but the magnitude of the change in elastic stretchability was relatively small compared to the elastic stretchability that the structure could achieve. In addition, the elastic stretchability of the kirigami structure increases with the number of periodic cuts, and the effect of increasing the number of periodic cuts on the elastic stretchability of the structure slightly decreases as the width of the cut increases.

The elastic stretchability of the structure versus spacing between the cuts and thickness of the substrate are shown as Figure 4b,c, respectively. It can be seen from the figure that for a certain number of periodic cuts, the elastic stretchability of the kirigami structure decreases with increasing spacing between the cuts and thickness of the substrate, and its effect on the elastic stretchability of the kirigami structure is more obvious. Additionally, the effect of the length of the cut on the elastic stretchability of the structure can be seen from Figure 4d. It can be concluded that for a certain number of periodic cuts, the elastic stretchability of the kirigami structure increases with the increase in the length of the cut, and the enhancement effect is obvious. For a certain length of the cut, when the cutting length is small, the increase in the number of periodic cuts has a negligible enhancement effect on the elastic stretchability of the structure, and this enhancement effect will gradually increase as the length increases.

### 3.3. Maximum Out-of-Plane Displacement Versus Geometric Parameters

This section mainly studies the influence of the geometric parameters of the kirigami structure on the out-of-plane maximum displacement. The size of the out-of-plane maximum displacement has a great influence on the size of the electronic device, and a smaller out-of-plane displacement maximum is beneficial to the packaging of the electronic device and has a great advantage in the miniaturization of the electronic device.

**Figure 5:**
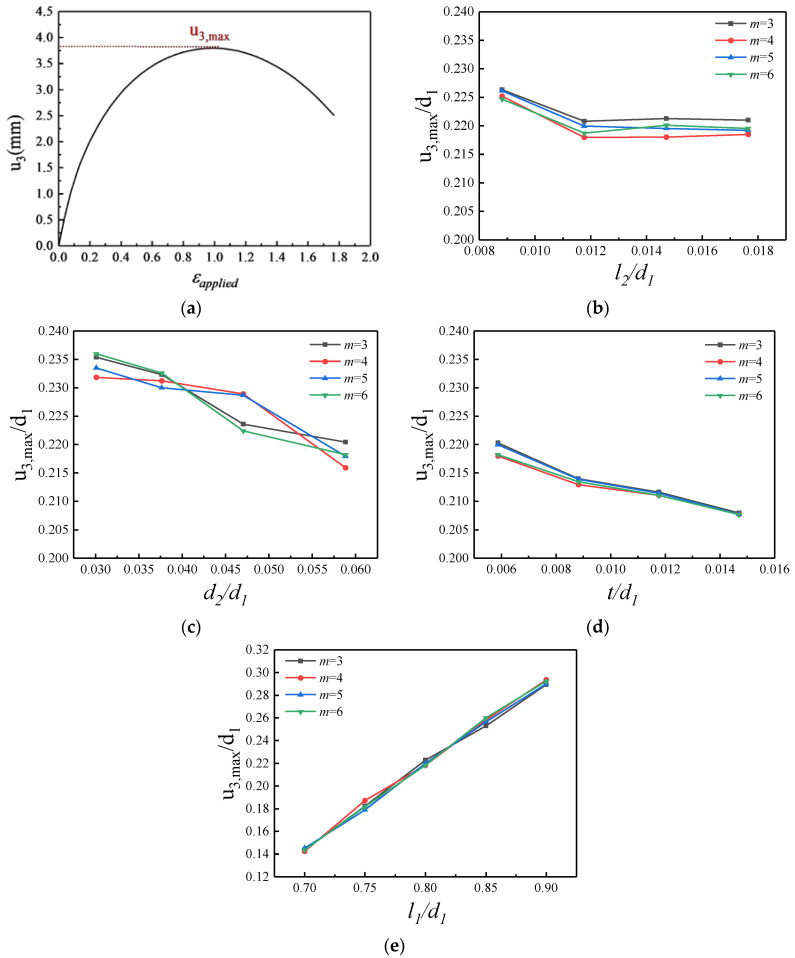
Theoretical and finite element results of maximum out-of-plane displacement. (**a**) Variation trend of out-of-plane displacement during tensile loading, (**b**) normalized cut width, (**c**) normalized spacing between the cuts, (**d**) normalized thickness of substrate, and (**e**) normalized cut length. The baseline geometric parameters are $l_{2}=0.2\;\mathrm{mm}$, $d_{2}=1\;\mathrm{mm}$, t=0.1 mm, and $l_{1}=13.6\;\mathrm{mm}$, and the parameter in each figure varies in its representative range.

During the stretching process of the kirigami structure, the out-of-plane displacement $u_{3}$ first increases and then decreases with the increase in the applied strain $\varepsilon_{\mathrm{applied}}$. The variation curve of the out-of-plane displacement during the loading process is shown in Figure 5a. When the applied strain ratio is small, the kirigami film mainly undergoes in-plane stretching, and the out-of-plane displacement increases from zero. As the applied strain continued to increase, the deformation mode of the kirigami structure changed from stretching to the bending and rotation of the strips. At this time, the out-of-plane displacement of the rotationally deformed structure of the strip reaches a maximum value, and then with the further increase in the applied strain, the deformation mode changes to tensile deformation parallel to the loading direction, and the out-of-plane displacement begins to gradually decrease.

The effect of cut width on the maximum out-of-plane displacement of the structure is shown in Figure 5b, selecting the dimensionless $l_{2}/d_{1}$ as the abscissa, where $l_{1}=13.6\;\mathrm{mm}$, $d_{1}=17\;\mathrm{mm}$, $d_{2}=1\;\mathrm{mm}$, and t=0.1 mm remain constant. It can be seen from the figure that when the width of the cut increases from 0.15 mm to 0.20 mm, the maximum out-of-plane displacement decreases slightly, and when the width of the cut continues to increase, the maximum out-of-plane displacement tends to be gentle and is smaller near a certain value. It can be concluded that when the width of the cut is small, its influence on the maximum out-of-plane displacement is relatively small and decreases with the increase in the width of the cut. And when the width of the cut continues to increase, the effect of the maximum out-of-plane displacement is small and can be ignored. In addition, when the number of periodic cuts changes, the maximum out-of-plane displacement has a small variation range, so the change in the number of periodic cuts has little effect on the maximum out-of-plane displacement.

Additionally, the maximum out-of-plane displacement in the structure versus the spacing between the cuts and thickness of the substrate are shown as Figure 5c,d, respectively. It can be seen from the figure that with the increase in the spacing between the cuts and thickness of the substrate, the maximum out-of-plane displacement of the kirigami structure decreases, but the decrease is smaller relative to the overall. Finally, the effect of the length of the cut on the maximum out-of-plane displacement can be seen from Figure 5e. The change in the length of the cut has a great influence on the maximum out-of-plane displacement of the kirigami structure. When the length of the cut increases, the maximum out-of-plane displacement will increase significantly.

### 3.4. Critical Buckling Load Versus Geometric Parameters

In this section, the critical buckling load for the buckling of the kirigami structure is studied. The eigenvalues corresponding to the buckling mode are obtained by solving the buckle analysis step in the finite element software ABAQUS. The critical buckling load for the kirigami structure buckling can be obtained by multiplying the eigenvalues by the load applied to the kirigami structure.

**Figure 6:**
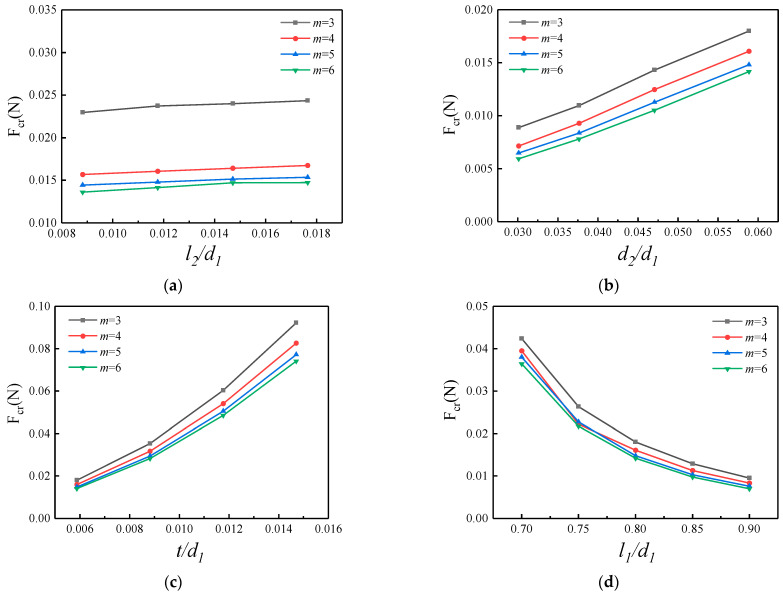
Theoretical and finite element results of critical buckling load. (**a**) Normalized cut width, (**b**) normalized spacing between the cuts, (**c**) normalized thickness of substrate, and (**d**) normalized cut length. The baseline geometric parameters are $l_{2}=0.2\;\mathrm{mm}$, $d_{2}=1\;\mathrm{mm}$, t=0.1 mm, and $l_{1}=13.6\;\mathrm{mm}$, and the parameter in each figure varies in its representative range.

The effect of cut width on the critical buckling load of the structure is shown in Figure 6a, selecting the dimensionless $l_{2}/d_{1}$ as the abscissa, where $l_{1}=13.6\;\mathrm{mm}$, $d_{1}=17\;\mathrm{mm}$, $d_{2}=1\;\mathrm{mm}$, and t=0.1 mm remain constant. It can be seen from Figure 6a that for a certain number of periodic cuts, the critical load changes less with the increase in the width of the cut. In addition, the critical buckling load decreases with the increase in the number of periodic cuts, and the decrease is more pronounced when the number of periodic cuts increases from three to four times. After that, increasing the number of periodic cuts has less effect on the critical buckling load.

The critical buckling load in the structure versus spacing between the cuts and thickness of the substrate are shown as Figure 6b,c, respectively. It can be seen from the figure that for a certain number of periodic cuts, with the increase in the spacing between the cuts and thickness of the substrate, the critical buckling load in the kirigami structure will increase. The thickness of the substrate has a more significant effect on the critical buckling load. In addition, an increase in the number of periodic cuts leads to a decrease in the critical buckling load. With the increase in the number of periodic cuts, this effect gradually decreases. Then, the effect of the length of the cut on the critical buckling load can be seen in Figure 6d. It can be concluded that the critical buckling load decreases with the increase in the length of the cut, and its decreasing effect is obvious. In addition, the critical buckling load decreases when the number of periodic cuts increases, but this effect is small and can be ignored.

### 3.5. Deformation of Kirigami Structures Under Bending and Torsion

This section mainly studies the maximum strain of the kirigami structure under the action of bending and torsion through the finite element method, and briefly evaluates its ability to withstand bending and torsional deformation.

**Figure 7:**
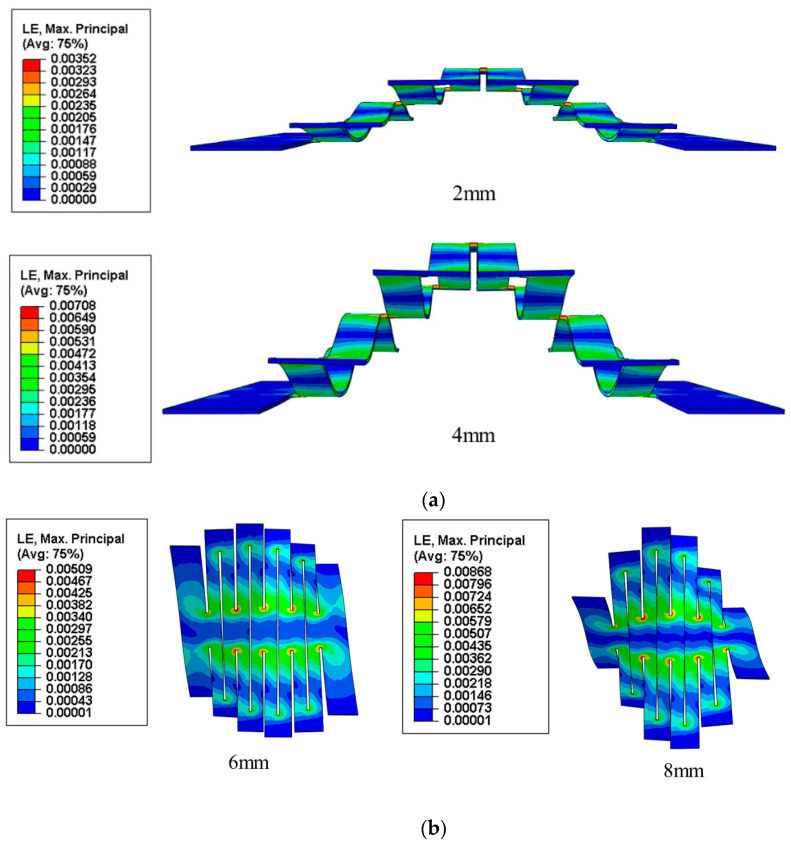
Strain nephogram of kirigami structure. (**a**) Bending deformation and (**b**) torsional deformation.

The strain nephogram of the kirigami structure under bending is shown in Figure 7a. A fixed load is applied at both ends of the kirigami structure and a downward displacement load is applied in the middle with displacement loads of 2 mm and 4 mm, where $l_{1}=13.6\;\mathrm{mm}$, $l_{2}=0.2\;\mathrm{mm}$, $d_{1}=17\;\mathrm{mm}$, $d_{2}=1\;\mathrm{mm}$, m=4, and t=0.1 mm remain constant.

It can be seen from the Figure 7a that the maximum strain in bending of the kirigami structure at a displacement load of 2 mm is about 0.35%, and the maximum strain in the structure when the displacement load is increased to 4 mm is only 0.71%, which is much smaller than the fracture strain of the material. It shows that the structure can maintain its integrity when subjected to a bending load, which in turn ensures stable electrical performance.

The strain nephogram of the kirigami structure under torsional deformation is shown in Figure 7b. The middle of the kirigami structure is fixed, and displacement loads in opposite directions are applied to both ends. It can be concluded that at a displacement load of 6 mm, the maximum strain is 0.51%, and when the applied displacement load is increased to 8 mm, the maximum strain increases to 0.87%, which is still much less than the fracture strain of the material. This indicates that the kirigami structure does not fail when subjected to a certain torsional load, thus maintaining the stability of the electronic device.

### 3.6. Conclusions

In this paper, the buckling behavior of kirigami structures under tension is studied by combining theory and finite element methods. The structure mainly realizes the ductility of electronic devices by making periodic incisions on flexible substrates. The influence of the geometric parameters of the kirigami structure (including cut length, cut width, cut spacing, number of cuts, and thickness of substrate) on the maximum strain, elastic stretchability, maximum out-of-plane displacement, and critical buckling load in the structure is mainly studied. Then, the maximum deformation of the kirigami structure under bending and torsion is briefly analyzed. Through the research and analysis, the following conclusions can be obtained:

(1) It can be concluded from the results of the study of the maximum strain that the effect of the cut width $l_{2}$ on the maximum strain of the structure is very small and negligible, and the maximum strain basically doesn’t change with it; when the cut spacing $d_{2}$ and the thickness of the substrate t are increased, the maximum strain in the structure increases approximately linearly with it; cut length $l_{1}$ has a large effect on the maximum strain of the structure, and the maximum strain decreases with the increase, and the two are no longer in a linear relationship; the effect of the number of cuts m on the maximum strain of the structure is relatively small, and the maximum strain decreases with the increase in the number of units, and the relationship is also no longer linear. In addition, as the applied strain $\varepsilon_{applied}$ increases, the maximum strain in the structure also increases.
(2) From the results of the structural elastic stretchability study, it can be concluded that cut width $l_{2}$ has a small effect on the structural stretchability, and the stretchability decreases by a small amount when it increases; cut spacing $d_{2}$ and thickness of the substrate t have a large effect on the structural elastic stretchability, and the stretchability decreases by a large amount when its value increases; cut length $l_{1}$ has the most significant effect on the structural stretchability, and the ductility increases by a very significant amount when its value increases; In addition, the increase in the number of cuts also improves the stretchability of the structure.
(3) From the results of the maximum out-of-plane displacement, it can be concluded that, except for cut length $l_{1}$, cut width $l_{2}$, cut spacing $d_{2}$, and thickness of the substrate t have less effect on the maximum out-of-plane displacement. In addition, the change in the number of cells hardly affects the maximum out-of-plane displacement. The above findings can provide some theoretical guidance for the size design of flexible electronic devices and the packaging strategy of the devices.
(4) From the results of the critical buckling load study, it can be concluded that the critical load of structural buckling is basically unaffected by cut width $l_{2}$; with the increase in the cut spacing $d_{2}$ and thickness of the substrate t, the critical buckling load also increases, and the increase in the thickness of the substrate t has a more obvious effect on the critical buckling load compared with cut spacing $d_{2}$; the critical buckling load decreases with the increase in cut length $l_{1}$, and the magnitude of the decrease is relatively obvious. Finally, for a fixed geometric parameter, the critical buckling load decreases with the increase in the number of cells, and this effect decreases with the increase in the number of cells.
(5) The kirigami structures can withstand certain bending and torsion loads in addition to large tensile loads.

The kirigami structure in this paper can significantly improve the ductility of electronic devices while having little effect on the electrical properties of electronic devices, and it has broad application prospects in the field of stretchable electronic devices.

## Data Availability

The original contributions presented in the study are included in the article, further inquiries can be directed to the corresponding author.
